# A Novel Paired Somatosensory-Cerebellar Stimulation Induces Plasticity on Cerebellar-Brain Connectivity

**DOI:** 10.1007/s12311-023-01622-5

**Published:** 2023-10-28

**Authors:** Francesca Ginatempo, Nicoletta Manzo, Danny A. Spampinato, Nicola Loi, Francesca Burgio, John C. Rothwell, Franca Deriu

**Affiliations:** 1 Department of Biomedical Sciences, University of Sassari, Viale San Pietro 43/b, 07100 Sassari, Italy; 2 IRCCS San Camillo, Via Alberoni, 70 Venice, Italy; 3 Department of Clinical and Behavioral Neurology, IRCCS Santa Lucia Foundation, Rome, Italy; 4 Department of Human Neurosciences, Sapienza University of Rome, Rome, Italy; 5 Sobell Department of Motor Neuroscience and Movement Disorders, UCL Institute of Neurology, London, UK; 6 Unit of Endocrinology, Nutritional and Metabolic Disorders, AOU, Sassari, Sassari Italy

**Keywords:** Cerebellar stimulation, Transcranial magnetic stimulation, Peripheral stimulation, Paired associative stimulation, Long-term depression

## Abstract

The cerebellum receives and integrates a large amount of sensory information that is important for motor coordination and learning. The aim of the present work was to investigate whether peripheral nerve and cerebellum paired associative stimulation (cPAS) could induce plasticity in both the cerebellum and the cortex. In a cross-over design, we delivered right median nerve electrical stimulation 25 or 10 ms before applying transcranial magnetic stimulation over the cerebellum. We assessed changes in motor evoked potentials (MEP), somatosensory evoked potentials (SEP), short-afferent inhibition (SAI), and cerebellum-brain inhibition (CBI) immediately, and 30 min after cPAS. Our results showed a significant reduction in CBI 30 minutes after cPAS, with no discernible changes in MEP, SEP, and SAI. Notably, cPAS10 did not produce any modulatory effects on these parameters. In summary, cPAS25 demonstrated the capacity to induce plasticity effects in the cerebellar cortex, leading to a reduction in CBI. This novel intervention may be used to modulate plasticity mechanisms and motor learning in healthy individuals and patients with neurological conditions.

## Introduction

Targeting the cerebellum with neuromodulatory non-invasive brain stimulation techniques has rapidly expanded over the past few decades, due to its unique anatomical structure and significant functional role in behavior [1]. Its densely packed population of neurons in the cerebellar cortex, intertwined with various closed-loop circuits, positions the cerebellum as a candidate for neuromodulation applications spanning a wide spectrum of motor and cognitive functions, including the treatment of neurological disorders. The ongoing pursuit of refining stimulation parameters and comprehending the ramifications of non-invasive cerebellar stimulation on plasticity and functional connectivity is vital to advancing the field of cerebellar neuromodulation [1].

Cerebellar plasticity is critical for integrating human motor control, learning, and memory, along with its capacity to influence communication with the primary motor cortex (M1) [1, 2]. Animal studies have provided evidence that the cerebellum plays a pivotal role in processing sensory feedback generated during movements and interactions with the surrounding environment, which not only enhances motor control but also facilitates the acquisition of motor skills [3–5]. In humans, non-invasive brain stimulation has also shown that increasing cerebellar excitability abolishes plasticity induced by peripheral nerve stimulation [6, 7]. This finding importantly shows the critical role of the cerebellum in priming M1 plasticity, potentially through the processing of sensory information [1]. Despite this evidence, no studies have attempted to develop plasticity protocols aimed at targeting somatosensory inputs that intervene in cerebellar and cortical pathways.

In a recent study, Bonassi et al. (2021) demonstrated that peripheral somatosensory inputs can influence cerebellar-motor cortex interaction, specifically cerebellar-brain inhibition (CBI), through a paired-pulse TMS protocol probing the cerebellar-M1 connectivity. Their findings indicated that delivering a peripheral electrical stimulus 25 and 35 ms prior to the TMS cerebellar stimulus reduced CBI, suggesting that somatosensory inputs caused changes in the activation of cerebellar interneurons and Purkinje cells, leading to the decreased CBI response [8]. Based on these results, we designed a new cerebellar paired associative stimulation (cPAS) protocol that required repeatedly applying specific timing intervals between the peripheral electrical stimulus and TMS cerebellar stimulus to induce plastic changes. This study specifically investigated the effect of cPAS applied at an interstimulus interval of 25 ms on CBI, M1 and S1 excitability, and sensorimotor integration.

## Methods

Twenty-one healthy right-handed volunteers were initially recruited for the study. However, one subject was excluded due to the low threshold for pyramidal tract activation by cerebellar stimulus, leaving a final sample size of 20 participants (12 females; mean age 28.7±1.3 years).

The sample size calculation was based on a post-hoc analysis using G*Power 3.1 software, assuming an expected effect size (Cohen’s *d*) of 0.7 in a single group recording at three different time points, resulting in a 0.05 alpha level and a total sample size of 20 to achieve a statistical power of 0.80.

The experimental procedure was approved by the local ethical committee and conducted in accordance with the Helsinki Declaration. All participants gave written consent to participate in the study. None of the participants had a history of neuro-psychiatric diseases or had taken drugs that could interfere with central nervous system activity.

### EMG

EMG was recorded from the right first dorsal interosseus (FDI) muscle using 9-mm diameter Ag-AgCl surface electrodes. The active electrode was placed over the muscle belly, the reference electrode at the second finger metacarpophalangeal joint, and the ground electrode over a forearm bony prominence, as previously described [9]. Unrectified EMG signals were recorded (D360, Digitimer Ltd, Welwyn Garden City, UK), amplified (×1000), filtered (bandpass 3–3000 Hz), and sampled (5 kHz) using a 1401 power analog-to-digital converter (Cambridge Electronic Design, Cambridge, UK) and Signal-5 software on a computer and stored for off-line analysis.

### SEP

The SEP was recorded following a previous clinical recommendation [10], with the median nerve stimulated using adhesive electrodes placed at the wrist (with the cathode positioned distal). These electrodes were connected to a constant-current stimulator (DS7; Digitimer Ltd.). To ensure that the position of the electrodes was correct, the muscle twitch on the thenar eminence was verified. Stimuli consisted of square wave pulses (0.2 ms duration; 3 Hz frequency; 600 trials; intensity able to evoke a muscle twitch). The N20–P25 waves were recorded by using an active electrode placed at CP3 and a reference electrode placed at Fz on the scalp, following the 10–20 international EEG system. The recorded signal was filtered with a bandpass of 3 Hz to 2 kHz and sampled at a rate of 5 kHz^10^. The peak-to-peak amplitude of the N20–P25 peak-to-peak wave was measured.

### TMS

TMS was delivered using a figure-of-eight coil with an external loop diameter of 7 cm, connected to a Magstim 200 stimulator (Magstim Co., Whitland, Dyfed, UK). The location of the “hot spot” over the FDI M1 representation was carefully identified and marked to maintain the same coil position throughout the experiments. The coil was positioned at 45° angle away from the midline, directed backward and laterally, as reported in previous works [11]. The resting motor threshold (RMT) was determined as the lowest TMS intensity that elicited MEPs of 0.05 mV in at least 5 out of 10 consecutive trials [11].

CBI was measured using a standard paired-pulse TMS paradigm [12–14]. Cerebellar stimulation (conditioning stimulus (CS)) was delivered by a double cone coil with an external loop diameter of 7 cm, positioned 3 cm lateral to the inion and contralaterally to M1. To avoid potential artifacts arising from antidromic stimulation of the pyramidal tract, the CS intensity was set at 60% MSO. This precaution was taken by verifying the brainstem activation threshold as described by previous studies [12, 14]. Subjects that showed pyramidal tract activation at 60% of MSO were excluded from the study [15, 16]. Only one recruited subject demonstrated clear pyramidal tract activation and was subsequently excluded from the study. The intensity of the test stimulus (TS) was set at 120% of RMT, and the interstimulus interval (ISI) between the CS and TS was set at 5 ms^16^.

SAI was obtained by pairing an electrical stimulus applied over the right median nerve (CS), using the same parameters as described for the SEP recording. This was paired with a TMS stimulus (TS) delivered to the contralateral M1, with an ISI of 20 ms [17, 18]. The intensity of the TS intensity was set at 120% RMT. For both CBI and SAI, twelve MEPs were obtained with the TS alone as done in a previous work [19], and twelve MEPs conditioned responses for each ISI were recorded in a randomized order. The test MEP was the same for both SAI and CBI protocols. SAI and CBI were quantified as the ratio between the amplitude of the conditioned MEP and unconditioned MEP.

### Experimental Design

The effect of different ISIs of cPAS was evaluated in two experimental sessions performed 2 weeks apart. An additional control experiment was performed only with cPAS25, with at least 1 week separating it from the main experiment.

#### Experiment 1. Effects of cPAS Intervention at ISIs of 25 ms (cPAS25) and 10 ms (cPAS10) on the Excitability of M1, S1, CBI, and SAI

The cPAS intervention was administered by pairing electrical stimulation of the right median nerve at an intensity three times the subjective perceptual threshold with a cerebellar TMS-pulse set at an intensity of 60% MSO, with an ISI of 25 ms. A total of two hundred pairs of stimuli were administered. MEPs, SEPs, CBI, and SAI were assessed before (baseline), 0 (T0), and 30 min (T30) after cPAS delivery.

In a separate experimental session, the cPAS protocol was delivered as described for cPAS25, but with a 10 ms ISI between ES and cerebellar TMS-pulse, serving as a control condition.

#### Experiment 2. Effects of cPAS25 on F-wave

To understand the effects of the cPAS25 on the spinal cord, F-waves were recorded in 10 out of 21 participants who participated in experiment 1. Twenty F-waves were collected from the right FDI following subjective supramaximal intensity stimulation of the ulnar nerve at the wrist, which was capable of evoking a compound muscle action potential (CMAP) with the maximum amplitude in the FDI muscle. F-wave persistence was used as a variable and expressed as the ratio between the number of F-wave detectable (amplitude >20 μV) and the total number of recordings [20]. The effects of cPAS25 were assessed by comparing F-wave persistence at baseline, immediately after cPAS25 (T0), and 30 min after cPAS25 (T30).

### Statistical Analysis

Data analysis for the MEP, CBI, and SAI protocols utilized the peak-to-peak MEP amplitude. Storing and offline MEP analysis were conducted using Signal 5.0 software on a computer. The ratios of conditioned MEP amplitude to unconditioned MEP amplitude were calculated for both SAI and CBI protocols [10, 15]. Th peak-to-peak N20 SEP amplitude was also measured as a variable measured using Signal 5 software.

Statistical analysis was performed with SPSS 20 software (SPSS Inc., Chicago, IL, USA). All variables were first tested for normality by using the Kolmogorov-Smirnov test. Student’s paired *t*-test, repeated measures analysis of variance (RM-ANOVA), and planned post hoc *t*-test with Bonferroni correction for multiple comparisons were used. The compound symmetry of data was evaluated using Mauchly’s test, and when necessary, Greenhouse-Geisser correction was applied. Statistical significance was set at a *p*-value <0.05. The results are presented as mean ± standard error of the mean (SEM).

#### Experiment 1

To evaluate whether significant inhibition occurred at baseline in the CBI and SAI protocols and to determine if differences occurred between sessions, a preliminary ANOVA was performed using raw amplitude MEP at baseline as a variable. Consequently, a two-way RM-ANOVA was performed, with cPAS (cPAS10 and cPAS25) and ISI (test MEP; conditioned MEP for CBI and SAI) as within-subjects factors.

Separate two-way RM-ANOVAs were performed on the raw amplitudes MEPs, raw amplitudes of SEPs, and MEP ratios (SAI and CBI) using cPAS (cPAS25, cPAS10) and TIME (baseline, T0, and T30) as within-subjects factors.

#### Experiment 2

For the evaluation of F-wave persistence, a one-way RM-ANOVA was performed using TIME (baseline, T0, and T30) as the within-subjects factor.

## Results

*Experiment 1. Effects of cPAS Intervention at ISIs of 25 ms (cPAS25) and 10 ms (cPAS10) on the Excitability of M1, S1, CBI, and SAI*

All variables were found to be normally distributed (all *p>0.05*). The raw amplitudes of the test and conditioned MEPs are reported in Table 1.

**Table 1:** Neurophysiological parameters

	cPAS10			cPAS25		
Raw amplitude (mV)	Before	T0	T30	Before	T0	T30
*Test MEP*	1.69±0.22	1.38±0.14	1.63±0.18	1.56±0.18	1.59±0.18	1.84±0.15
*Conditioned MEP in CBI protocol*	1.33±0.23	1.11±0.16	1.29±0.14	1.02±0.15	1.29±0.22	1.73±0.19
*Conditioned MEP in SAI protocol*	1.29±0.36	1.20±0.34	1.30±0.31	0.80±0.17	0.83±0.16	1.26±0.21

*MEP* motor evoked potentials, *CBI* cerebellar-brain inhibition, *SAI* short afferent inhibition. The table reports mean ±standard mean error (SEM)

### MEP

RM-ANOVA showed a significant effect of TIME (*F*_1,19_=1.175; *p=0.319*) but a non-significant effect of cPAS (*F*_1,19_=0.532; *p=0.476*), and no significant interaction between factors (*F*_1,19_=0.299; *p=0.694*).

### SEP

RM-ANOVA showed a non-significant effect of cPAS (*F*_1,19_=1.65; *p=0.217*), TIME (*F*_1,19_=1.192; *p=0.309*), and no significant interaction among factors (*F*_1,19_=2.994; *p=0.07*).

### CBI and SAI

Preliminary analysis failed to detect any significant difference at baseline between the two sessions but revealed a significant inhibition in both the CBI and SAI protocols. The two-way RM-ANOVA showed a significant effect of ISI (*F*_1,19_=22.785; *p<0.001*) but a non-significant effect of cPAS (*F*_1,19_=0.450; *p=0.511*) and no significant interaction between factors (*F*_1,19_=0.399; *p=0.660*). Post hoc analysis revealed a reduced conditioned MEP following paired stimulation than test MEP (CBI: *p=0.005*; SAI: *p=0.009*).

### CBI

**Fig. 1:**
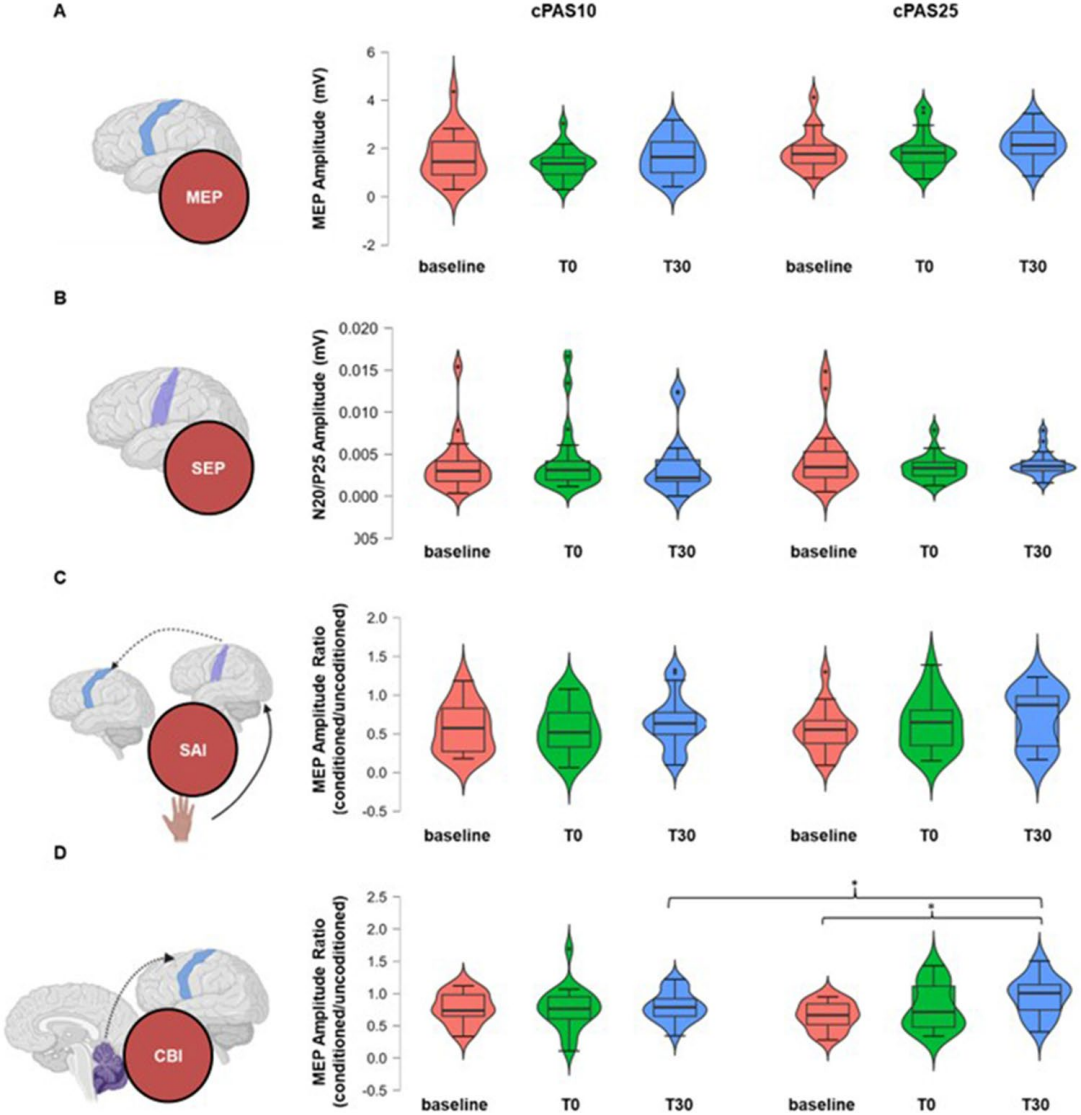
Effects of cPAS25 and cPAS10 on excitability of primary somatosensory and motor cortices, sensorimotor integration, and cerebellar-brain inhibition. The violin plots report motor evoked potential (MEP) (**A**), somatosensory evoked potentials (SEP) (**B**), short afferent inhibition (SAI) (**C**), and cerebellar-brain inhibition (CBI) (**D**) before and after 0 (T0) and 30 min (T30) from delivery of cPAS10 or cPAS25. Ordinates indicate raw MEP amplitude (**A**), raw P20/N25 amplitude (**B**), and the MEP amplitude ratio (conditioned/unconditioned MEP) for SAI (**C**) and CBI (**D**). *p<0.05

cPAS10 did not induce any changes in CBI, while it was significantly reduced 30 min after cPAS25 (Fig. 1). RM-ANOVA showed a non-significant effect of cPAS (*F*_1,19_=0.335; *p=0.570*) but a significant effect of TIME (*F*_1,19_=4.625; *p=0.017*) and a significant interaction between factors (*F*_1,19_=4.218; *p=0.026*). Post hoc analysis on the TIME effect showed that CBI at T30 was significantly reduced compared with baseline (*p=0.016*). The Bonferroni test on the interaction revealed a significantly reduced CBI at T30 after cPAS25 (*p<0.001*), and differences in CBI in the two cPAS sessions were only observed at T30 (*p=0.042*).

### SAI

RM-ANOVA showed a non-significant effect of cPAS (*F*_1,19_=0.016; *p=0.902*), TIME (*F*_1,19_=1.832; *p=0.182*), and no significant interaction between factors (*F*_1,19_=0.516; *p=0.550*).

*Experiment 2. Effects of cPAS25 on F-wave*

One-way ANOVA did not reveal a significant effect of cPAS25 on F-wave persistence (*F*_2,16_=0.384; *p=0.662*).

## Discussion

The results of this study demonstrate that cPAS25 induces changes in cerebellar excitability, as reflected by the reduction of CBI observed 30 min post-intervention. This result suggests that this protocol may induce a potential plasticity effect, which appears to be specifically localized to the cerebellum, as there were no changes in M1, as indicated by the lack of changes in MEP amplitude following the intervention. This finding also suggests that alterations in cortical excitability are unlikely to account for the observed modulation of CBI. Additionally, the absence of changes in both SEP and SAI following cPAS25 implies that neither primary somatosensory cortex activity nor sensorimotor integration plays a role in the CBI reduction triggered by cPAS25. Finally, the stability of F-wave amplitude post-intervention suggests that alterations in spinal excitability are unlikely contributors.

Importantly, our control condition (cPAS10) failed to induce any plastic changes in either the cerebellum or cortex. This aligns with previous research showing that median SEP latency at the cervical cord is approximately 13 ms, making it unlikely for the stimulus in cPAS10 to reach the cerebellum [10].

### What Might Explain the Changes Seen in CBI Using cPAS25?

The sensory information elicited by median nerve stimulation in cPAS can travel through two distinct pathways, potentially leading to modifications in cerebellar function through mechanisms resembling spike-time-dependent plasticity-like mechanisms. These pathways include the spino-inferior olivary (IO) fasciculus and the spino-cuneo-cerebellar tract. Ultimately, both pathways convey excitatory signals to the cerebellar cortex and the cerebellar nuclei [1, 2, 12, 21, 22].

Of note, the IO fasciculus typically responds to unexpected stimuli that are not self-generated through active movements [23–27]. This feature of the IO fasciculus, coupled with the convergence of ascending peripheral and descending cortical inputs onto it, suggests the role of the IO-cerebellar complex as a detector for unexpected events, which could potentially modulate responses to peripheral inputs that were not anticipated [28–30]. In this context, the electrical stimulation in cPAS could be interpreted as an unexpected stimulus, activating olivo-cerebellar pathways, including excitatory connections to Purkinje cells via climbing fibers and climbing fiber collaterals to the deep cerebellar nuclei [2, 23–27]. The level of spontaneous Purkinje cell activity is thus regulated by the activity of the inferior olive. Since TMS over the cerebellum is thought to activate Purkinje cells [1, 2, 12–14], one possible explanation for the effects of cPAS25 may be related to a LTD effect on climbing fiber-Purkinje cell synapses, resulting in a reduction of CBI.

An alternative mechanism that may underlie the effects of cPAS25 on CBI can be explained by the sensory information entering the cerebellum through the spino-cuneo-cerebellar tract. This information enters the cerebellum via the mossy fibers, which are then processed in the granular layer and transmitted to Purkinje cells via parallel fibers, with a copy relayed in cerebellar nuclei [2, 23–27]. The intricate cerebellar circuitry of this pathway suggests that, apart from climbing fiber-Purkinje cell LTD, it is also possible that LTP between granule cells and inhibitory interneurons in the cerebellar cortex could contribute to a reduced CBI^27^. In summary, our findings suggest that the effects of cPAS25 on CBI may result from a combination of complex pathways and potential plasticity mechanisms within the complex neural circuitry of the cerebellum. Further investigations are needed to better understand the precise interplay of these mechanisms and their implications for cerebellar excitability and sensorimotor integration.

### Limitation of the Study

In the present study, only two ISIs were investigated to standardize cPAS protocol. However, future studies should consider including a broader range of ISIs to comprehensively evaluate this phenomenon.

## Conclusions

It is likely that cPAS25 represents a novel protocol capable of inducing plastic changes in the cerebellar cortex. As we have tested only a limited range of ISIs, future work should explore additional intervals to potentially yield more robust effects. Nevertheless, this protocol has the potential to modulate cerebellar plasticity in physiological and neurological conditions. Specifically, this protocol could help in assessing abnormal circuits of plasticity observed in neurological disorders and potentially contribute to the restoration of these abnormalities.

## Data Availability

The data that support the findings of this study are made available from the corresponding author, upon reasonable request.
